# A Note on the Spectral Gap of the Fredrickson–Andersen One Spin Facilitated Model

**DOI:** 10.1007/s10955-020-02666-1

**Published:** 2020-11-04

**Authors:** Assaf Shapira

**Affiliations:** Universita degli Studi Roma Tre, Rome, Italy

**Keywords:** Kinetically constrained models, Interacting particle systems, Relaxation time

## Abstract

This note discusses the spectral gap of the Fredrickson–Andersen one spin facilitated model in two different settings. The model describes an interacting particle system on a graph, where each site is either occupied or empty; and a site may change its occupation when at least one of its neighbors is empty. We will first consider the model on the infinite lattice ${\mathbb {Z}}^{d}$, with density close to 1. The second result is on finite graphs, with density that grows with the size of the graph in a way that guarantees *O*(1) empty sites. In both models lower and upper bounds on the spectral gap were known, but in general did not match. The purpose of this paper is to present new upper bounds that have the same asymptotics as the known lower bounds.

## Introduction and Results

We study the Fredrickson–Andersen one spin facilitated model (FA1f), on the lattice following [1] and on finite graphs following [3, 5, 6]. The reader is referred to [1, 3, 5, 6] for the relevant background, references, and complete introduction of the model.

We will only briefly remind here that sites in a graph *G* could be either occupied or empty, with equilibrium probabilities $1-q$ and *q* respectively (where *q* is thought of as small). When a site has at least one empty neighbor, it is being resampled from equilibrium with rate 1, and otherwise its occupation cannot change. This process is reversible with respect to a product measure conditioned on having at least one empty site, that we refer to as the *equilibrium measure*. The infinitesimal generator of the process is denoted by ${\mathcal {L}}_{G}$, and its spectral gap (with respect to the equilibrium measure) by ${\text {gap}}({\mathcal {L}}_{G})$. The inverse of the spectral gap, called the relaxation time, gives the typical time over which correlations are lost in stationarity.

The first result here completes Theorem 6.4 of [1], which bounds the spectral gap of the FA1f model when the graph *G* is the lattice ${\mathbb {Z}}^{d}$. It is shown there that the gap decays polynomially as the parameter *q* tends to 0, and for dimensions 1 and 2 the exact exponent is identified, up to a logarithmic correction in dimension 2. For dimension $d\ge 3$, however, the exponent is bounded between $1+2/d$ and 2, and its exact value is not determined. The following theorem shows that the correct scaling is $q^{2}$.

### Theorem 1.1

Consider the FA1f model on the graph $G={\mathbb {Z}}^d$ for $d\ge 3$. Then there exists a positive constant *C* (possibly depending on *d*) such that

$$\begin{aligned} {\text {gap}}({\mathcal {L}}_{{\mathbb {Z}}^{d}})\le C\,q^{2}. \end{aligned}$$

The second theorem that will be presented discusses the model on a finite graph *G*. A particularly interesting case, studied in [5, 6] and more recently in [3], is when $\left| V(G)\right| =cq^{-1}$ for some positive constant *c*, where *V*(*G*) denotes the set of sites in *G*. A lower bound on the spectral gap is given in [5, 6] and later on in [3] by suggesting a relaxation mechanism in which vacancies travel as random walkers on *G*. The next theorem bounds the spectral gap of this model from above, showing that this mechanism has a leading contribution. Consider two independent continuous time random walks on *G*, namely, each of the two walkers moves to each neighboring site with rate 1. For two vertices $x,y\in V(G)$, we define $\tau _{{\text {meet}}}(x,y)$ to be the expected time that it takes for two such random walkers starting at *x* and *y* to reach distance at most 1. Let $\overline{\tau }_{{\text {meet}}}$ be its expected value, starting at two uniform random positions, i.e., $\overline{\tau }_{{\text {meet}}}=\left| V(G)\right| ^{-2}\sum _{x,y\in V(G)}\tau _{{\text {meet}}}(x,y)$.

### Theorem 1.2

Consider the FA1f model on a finite graph *G*, and assume that $\left| V(G)\right| =c/q$ for some positive constant *c*. Then

$$\begin{aligned} {\text {gap}}({\mathcal {L}}_{G})\le \frac{Cq}{\overline{\tau }_{{\text {meet}}}} \end{aligned}$$

for some positive constant *C*.

### Remark 1.3

[3, Eqs. (4), (13)] give a lower bound on the spectral gap, which in various graphs is of the same order as the upper bound given in Theorem 1.2. In particular, on the two dimensional torus ${\mathbb {T}}^{2}={\mathbb {Z}}^{2}/\ell {\mathbb {Z}}^{2}$ with $\ell =cq^{-1/2}$, both the upper and lower bounds scale like $q^{2}/\log (1/q)$. In view of this scaling, and the relaxation mechanism reflected in its proof, it seems that the correct scaling of the spectral gap is $q^{2}/\log (1/q)$ also in ${\mathbb {Z}}^{2}$, coinciding with the lower bound of [1]. Unfortunately, the ideas in the proof of Theorem 1.2 do not seem to be easily adapted for the model on ${\mathbb {Z}}^{2}$, and the problem remains open.

## Notation

We will now introduce some notation that will be used in the proofs. See also [1].

- The FA1f dynamics is defined on a graph *G* with vertex set *V*(*G*). For $x,y\in V(G)$ we denote by $d_{G}(x,y)$ the graph distance between *x* and *y*.
- The configuration space is $\{0,1\}^{V(G)}$, and the equilibrium measure is denoted $\mu _{G}$. On infinite graphs $\mu _{G}$ is a product measure of Bernoulli random variables with parameter $1-q$. For finite graphs, $\mu _{G}$ is given by the same product, conditioned on having at least one empty site. This measure could also be seen as the unique invariant measure on the space $\Omega _G = \{0,1\}^{V(G)} \setminus \{\mathbf {1}\}$ with $\mathbf {1}$ being the fully occupied configuration.
- For $\eta \in \{0,1\}^{V(G)}$ and $x\in V(G)$, the configuration which equals $\eta$ outside *x* and different from $\eta$ at *x* is denoted $\eta ^{x}$.
- The constraint of the FA1f dynamics, for a configuration $\eta \in \{0,1\}^{V(G)}$ and $x\in V(G)$, is
  $$\begin{aligned} c_{x}(\eta )={\left\{ \begin{array}{ll} 1 &{} \exists y\text { such that }d(x,y)=1\text { and }\eta (y)=0,\\ 0 &{} \text {otherwise}. \end{array}\right. } \end{aligned}$$
- The Dirichlet form of FA1f operating on a local function $f:\Omega _{G}\rightarrow {\mathbb {R}}$ is given by
  $$\begin{aligned} {\mathcal {D}}_{G}(f)&=\sum _{x\in V(G)}\mu \left( c_{x}{\text {Var}}_{x}(f)\right) =q(1-q)\sum _{x\in V(G)}\mu \left( c_{x}(f(\eta )-f(\eta ^{x}))^{2}\right) . \end{aligned}$$
  At this point we also recall that the spectral gap has the following variational characterization:
  2.1 $$\begin{aligned} {\text {gap}}({\mathcal {L}}_G) = \inf _{f} \frac{{\mathcal {D}}_G(f)}{{\text {Var}}(f)}, \end{aligned}$$
  where the infimum is taken over all function on $\Omega _G$ which are not identically 0.

Throughout the proof *C* will denote a generic positive constant, and *q* is assumed to be small enough.

**Warning:** When context allows, we may omit the subscript *G* when referring to $d_{G},\Omega _{G},\mu _{G},{\mathcal {D}}_{G}$.

## Proof of Theorem 1.1

In this section we consider the graph $G={\mathbb {Z}}^{d}$ for $d\ge 3$. In order to bound the spectral gap from above, we need to find an appropriate test function $f:\Omega _{{\mathbb {Z}}^{d}}\rightarrow {\mathbb {R}}$, such that

$$\begin{aligned} {\mathcal {D}}(f)\le Cq^{2}\,{\text {Var}}(f). \end{aligned}$$

Consider the box $\Lambda =\{0,\dots ,\ell -1\}^{d}$ for $\ell =\left\lfloor {1/q}\right\rfloor$. For a configuration $\eta$ and a site $x\in \Lambda$, the *connected cluster of* *x*, denoted ${\mathcal {C}}_{x}(\eta )$, is defined as the set of sites $y\in \Lambda$ that are connected to *x* via a path of empty sites in $\Lambda$. If $\eta (x)=1$, its connected cluster is the empty set. This way, the set of empty sites in $\Lambda$ is partitioned in connected clusters, and we define:

3.1 $$\begin{aligned} f(\eta )=\#\text {non-empty connected clusters in }\Lambda . \end{aligned}$$

### Proposition 3.1

For the test function *f* defined in Eq. (3.1),

3.2 $$\begin{aligned} {\text {Var}}(f)\ge C\,q\,\ell ^{d}. \end{aligned}$$

### Proof

This result is shown in [2] for the case of Bernoulli bond percolation. We will repeat their argument applied to our case for completeness.

First, note that we may write

$$\begin{aligned} f(\eta )=\sum _{x\in \Lambda }\frac{1-\eta (x)}{|{\mathcal {C}}_{x}(\eta )|}, \end{aligned}$$

where, when $\eta (x)=1$ (and therefore ${\mathcal {C}}_{x}(\eta )=\emptyset$), we define $\frac{1-\eta (x)}{|{\mathcal {C}}_{x}(\eta )|}=0$.

Let $G_{3}=3{\mathbb {Z}}^{d}\cap \Lambda$, and for $A\subseteq G_{3}$, define $\chi _{A}(\eta )$ to be the indicator of the event, that the set $\{x\in G_3:\eta (y)=1\,\,\forall y\text { such that }d(x,y)=1\}$ is equal *A*. Note that

$$\begin{aligned} \mu (\chi _{A})=(1-q)^{2d|A|}\cdot \left( 1-(1-q)^{2d}\right) ^{|G_{3}|-|A|}. \end{aligned}$$

For such a set *A*, let $D(A)=\{y\in \Lambda :d(x,y)\le 1\text { for some }x\in A\}$, and define

$$\begin{aligned} f_{A}(\eta )=\sum _{x\in \Lambda \setminus D(A)}\frac{1-\eta (x)}{\left| {\mathcal {C}}_{x}(\eta )\right| }. \end{aligned}$$

When $\eta$ is such that $\chi _{A}(\eta )=1$,

$$\begin{aligned} f(\eta )=f_{A}(\eta )+\sum _{x\in A}(1-\eta (x))=: f_{A}(\eta )+n_{A}(\eta ). \end{aligned}$$

In order to use this identity, we split the variance over the different choices of *A*:

$$\begin{aligned} {\text {Var}}(f)=\sum _{A\subseteq G_{3}}\mu \left( (f-\mu (f))^{2}\chi _{A}\right) . \end{aligned}$$

Consider one of the summands in the above expression –

$$\begin{aligned} \mu \left( (f-\mu (f))^{2}\chi _{A}\right)&=\mu \left( (f_{A}-(\mu (f)+\mu (n_{A})) +n_{A}-\mu (n_{A}))^{2}\chi _{A}\right) \\&=\mu \left( (f_{A}-(\mu (f)+\mu (n_{A}))^{2}\chi _{A}\right) +\mu \left( (n_{A} -\mu (n_{A}))^{2}\chi _{A}\right) \\&\quad +\mu \left( (f_{A}-(\mu (f)+\mu (n_{A}))(n_{A}-\mu (n_{A}))\chi _{A}\right) . \end{aligned}$$

The first term is positive, and we will simply bound it by 0. In order to find the second term, we note that the variables $n_{A}$ and $\chi _{A}$ are independent, and therefore

$$\begin{aligned} \mu \left( (n_{A}-\mu (n_{A}))^{2}\chi _{A}\right)&=\mu (\chi _{A}){\text {Var}}(n_{A})\\&=(1-q)^{2d|A|} \cdot \left( 1-(1-q)^{2d}\right) ^{|G_3|-|A|}\cdot \left| A\right| q(1-q). \end{aligned}$$

Finally, since under the event $\{\chi _{A}=1\}$ the variables $f_{A}$ and $n_{A}$ are independent, the third term vanishes. Therefore,

$$\begin{aligned} {\text {Var}}(f)\ge \sum _{A\subseteq G}(1-q)^{2d|A|}\cdot \left( 1-(1-q)^{2d}\right) ^{|G_{3}|-|A|} \cdot \left| A\right| q(1-q)=q(1-q)^{2d+1}\left| G_{3}\right| . \end{aligned}$$

This establishes inequality (3.2). $\square$

### Proposition 3.2

For the test function *f* defined in Eq. (3.1),

3.3 $$\begin{aligned} {\mathcal {D}}(f)\le Cq^{3-d}. \end{aligned}$$

### Proof

Recall first that

$$\begin{aligned} {\mathcal {D}}(f)=q(1-q)\sum _{x\in {\mathbb {Z}}^{d}}\mu \left( c_{x}(\eta ) \left( f(\eta ^{x})-f(\eta )\right) ^{2}\right) . \end{aligned}$$

Consider a single term in that sum. First, note that by flipping a single site *f* could change by at most 2*d*. If *x* is outside $\Lambda$, flipping it could not change the number of clusters in $\Lambda$ and its contribution would be 0. If *x* is on the boundary of $\Lambda$ (i.e., it is in $\Lambda$ and has a neighbor outside $\Lambda$), then

$$\begin{aligned} \mu \left( c_{x}(\eta )\left( f(\eta ^{x})-f(\eta )\right) ^{2}\right) \le 4d^2 \mu \left( c_{x}(\eta )\right) \le C\,q. \end{aligned}$$

Finally, if *x* is in $\Lambda$ but has no neighbors outside $\Lambda$, the number of open clusters could only change if it has at least two empty neighbors:

$$\begin{aligned} \mu \left( c_{x}(\eta )\left( f(\eta ^{x})-f(\eta )\right) ^{2}\right) \le 4d^2 \,\mu (\mathbb {1}_{x\text { has at least }2\text { empty neighbors}})\le C\,q^{2}. \end{aligned}$$

The proof is now concluded by summing these options:

$$\begin{aligned} \sum _{x\in {\mathbb {Z}}^{d}}\mu \left( c_{x}(\eta )\left( f(\eta ^{x})-f(\eta )\right) ^{2}\right) \le C\ell ^{d-1}q+C\ell ^{d}q^{2}=Cq^{-d+2}. \qquad \qquad \qquad \qquad \qquad \qquad \square \end{aligned}$$

Theorem 1.1 follows from Eqs. (3.2) and (3.3), together with the variational characterization of the spectral gap. $\square$

## Proof of Theorem 1.2

As in the proof of Theorem 1.1, we look for $f:\Omega _{G}\rightarrow {\mathbb {R}}$ such that

$$\begin{aligned} {\mathcal {D}}(f)\le Cd_{\max }q\overline{\tau }_{{\text {meet}}}^{-1}\,{\text {Var}}(f), \end{aligned}$$

where the variance is understood with respect to the measure $\mu _{G}$. Recall that $\tau _{\mathrm{meet}}(x,y)$ is the expected meeting time of two random walkers on *G*, starting at positions *x* and *y*; and that its expected value for *x* and *y* chosen uniformly at random is denoted $\overline{\tau }_{{\text {meet}}}$. “Meeting” here means that their graph distance is at most 1.

The test function that we use is

4.1 $$\begin{aligned} f(\eta )=\max _{\begin{array}{c} x,y\in V(G)\\ \eta (x)=\eta (y)=0 \end{array}}\tau _{\mathrm{meet}}(x,y). \end{aligned}$$

Before analyzing the function *f*, note that $\tau _{\mathrm{meet}}$ solves the following Poisson equation:

4.2 $$\begin{aligned} -{\mathcal {L}}_{\mathrm{RW}}\left( \tau _{\mathrm{meet}}(x,y)\right)&=1,\qquad d(x,y)>1, \nonumber \\ \tau _{\mathrm{meet}}(x,y)&=0,\qquad d(x,y)\le 1; \end{aligned}$$

where ${\mathcal {L}}_{\mathrm{RW}}$ is the infinitesimal generator of two independent random walks on *G*. Multiplying both sides by $\tau _{\mathrm{meet}}(x,y)$ and averaging over *x* and *y* we obtain

4.3 $$\begin{aligned} \overline{\tau }_{\mathrm{meet}}={\mathcal {D}}_{\mathrm{RW}} \left( \tau _{\mathrm{meet}}\right) , \end{aligned}$$

where the Dirichlet form is given for every $g:V(G)\times V(G)\rightarrow {\mathbb {R}}$ by

$$\begin{aligned} {\mathcal {D}}_{\mathrm{RW}}\left( g\right) =\frac{1}{2\left| V(G)\right| ^{2}} \sum _{x}\sum _{y}\left[ \sum _{x'\sim x}\left( g(x',y)-g(x,y)\right) ^{2} +\sum _{y'\sim y}\left( g(x,y')-g(x,y)\right) ^{2}\right] . \end{aligned}$$

### Proposition 4.1

For *f* defined in Eq. (4.1),

$$\begin{aligned} {\text {Var}}(f)\ge C\overline{\tau }_{{\text {meet}}}^{2}. \end{aligned}$$

### Proof

Under $\mu ,$ the probability that exactly one site is empty is of order 1 (i.e., bounded away from 0 uniformly in *q*). When this happens, $f=0$, so

$$\begin{aligned} {\text {Var}}(f)&=\mu \left[ \left( f-\mu (f)\right) ^{2}\right] \ge \mu \left[ \left( f-\mu (f)\right) ^{2}\mathbb {1}_{\sum _{x}(1-\eta (x))=1}\right] \\&=\mu (f)^{2}\,\mu \left[ \sum _{x}(1-\eta (x))=1\right] \ge C\mu (f)^{2}. \end{aligned}$$

When a configuration has exactly two empty sites, *f* is given by the positions of these vacancies:

$$\begin{aligned} \mu \left[ f(\eta )\big |\mathbb {1}_{\sum _{x}(1-\eta (x))=2}\right] =\frac{2}{\left| V(G)\right| (\left| V(G)\right| -1)}\sum _{\begin{array}{c} x,y\in V(G)\\ x\ne y \end{array} }\tau _{\mathrm{meet}}(x,y)\ge \overline{\tau }_{\mathrm{meet}}. \end{aligned}$$

The result follows from these two estimates since the probability to have exactly two vacancies is of order 1. $\square$

### Proposition 4.2

For *f* defined in Eq. (4.1),

$$\begin{aligned} {\mathcal {D}}(f)\le C\,q\overline{\tau }_{{\text {meet}}}. \end{aligned}$$

### Proof

We start the proof with an observation:

### Observation 4.3

Let $\eta \in \Omega$ and $x\in V(G)$ such that $c_{x}(\eta )=1$, $\eta (x)=0$, and $f(\eta )\ne f(\eta ^{x})$. Then there exist $x',y\in V(G)$ such that $x'\sim x$, $d(x,y)>1$, $\eta (y)=\eta (x')=0$, and

$$\begin{aligned} \tau _{{\text {meet}}}(x',y)\le f(\eta ^{x})<f(\eta )=\tau _{{\text {meet}}}(x,y). \end{aligned}$$

### Proof

First, recalling Eq. (4.1), when filling an empty site *f* could only decrease, and since $f(\eta )\ne f(\eta ^{x})$ necessarily $f(\eta )>f(\eta ^{x})$. Moreover, *f* could only change if the maximum is attained at the pair *x*, *y* for some $y\in V(G)$, i.e., $f(\eta )=\tau _{\mathrm{meet}}(x,y)$. Note that $f(\eta )$ is non-zero, hence $d(x,y)>1$. Finally, $c_{x}(\eta )=1$ means that *x* has an empty neighbor $x'$; and since in the configuration $\eta ^{x}$ both $x'$ and *y* are empty $f(\eta ^{x})\ge \tau _{\mathrm{meet}}(x',y)$. $\square$

As a consequence of this observation, for all $\eta \in \Omega$ and *x* such that $c_{x}(\eta )=1$,

$$\begin{aligned} \left( f(\eta ^{x})-f(\eta )\right) ^{2}\le \sum _{\begin{array}{c} y\in V(G)\\ d(x,y)>1 \end{array}}\sum _{\begin{array}{c} x'\in V(G)\\ x'\sim x \end{array}}(1-\eta (y))(1-\eta (x')) \,\left( \tau _{\mathrm{meet}}(x,y)-\tau _{\mathrm{meet}}(x',y)\right) ^{2}. \end{aligned}$$

We can now use this estimate and calculate the Dirichlet form:

$$\begin{aligned} {\mathcal {D}}(f)&=q(1-q)\sum _{x}\mu _{G}\left[ c_{x}\left( f(\eta ^{x}) -f(\eta )\right) ^{2}\right] \\&\le q(1-q)\sum _{x}\mu _{G}\left[ \sum _{\begin{array}{c} y\in V(G)\\ d(x,y)>1 \end{array} }\sum _{x'\sim x}(1-\eta (y))(1-\eta (x'))\,\left( \tau _{\mathrm{meet}}(x,y) -\tau _{\mathrm{meet}}(x',y)\right) ^{2}\right] \\&\le q^{3}(1-q)\sum _{x}\sum _{\begin{array}{c} y\in V(G)\\ d(x,y)>1 \end{array}}\sum _{x'\sim x}\left( \tau _{\mathrm{meet}}(x,y) -\tau _{\mathrm{meet}}(x',y)\right) ^{2} \le Cq\,{\mathcal {D}}_{\mathrm{RW}}(\tau _{\mathrm{meet}}), \end{aligned}$$

and the proposition follows from Eq. (4.3). $\square$

Theorem 1.2 is a consequence of Propositions 4.1 and 4.2, using the variational characterization of the spectral gap. $\square$

### Remark 4.4

For the case of the two dimensional torus discussed in Remark 1.3, it is possible to show that $\overline{\tau }_{\mathrm{meet}}$ is greater than $Cq^{-1}\log (1/q)$, obtaining an upper bound on the spectral gap which matches the lower bound of [3]. One way to do that is by comparing it to $\tau _{{\text {meet}}}^{0}(x,y)$, the expected time required for two random walkers to arrive at *the same* site, starting at *x* and *y*. Note that in the case of the torus this is half the time a single walker starting at *x* takes to reach *y*. In [4, Section 10.4], for example, it is shown that there exist two positive constants $c_{1},c_{2}$ such that

$$\begin{aligned} c_{1}q^{-1}\,\log (d(x,y))\le \tau _{{\text {meet}}}^{0}(x,y)\le c_{2}q^{-1}\,\log (d(x,y)+1). \end{aligned}$$

In particular, $\left| V(G)\right| ^{-2}\sum _{x,y}\tau _{{\text {meet}}}^{0}(x,y)$ is of order $q^{-1}\log (1/q)$, and $\max _{x\sim y}\tau _{{\text {meet}}}^{0}(x,y)\le Cq^{-1}$. The last estimate implies that $\tau _{{\text {meet}}}^{0}$ satisfies

$$\begin{aligned} -{\mathcal {L}}_{\mathrm{RW}}\left( \tau _{\mathrm{meet}}^{0}(x,y)-Cq^{-1}\right)&=1,\qquad d(x,y)>1,\\ \tau _{\mathrm{meet}}^{0}(x,y)-Cq^{-1}&\le 0,\qquad d(x,y)\le 1. \end{aligned}$$

That is, the function $\tau _{\mathrm{meet}}^{0}(x,y)-Cq^{-1}$ it is a sub-solution of Eq. (4.2). Hence by the maximum principle, $\tau _{{\text {meet}}}(x,y)\ge \tau _{\mathrm{meet}}^{0}(x,y)-Cq^{-1}$ for all *x*, *y*. In particular, $\overline{\tau }_{{\text {meet}}}$ is at least $Cq^{-1}\log (1/q)$.
